# Hypertrophic Cardiomyopathy

**DOI:** 10.3390/jcdd10030106

**Published:** 2023-03-02

**Authors:** Asra K. Butt, Deya Alkhatib, Issa Pour-Ghaz, Sakiru Isa, Omar Al-Taweel, Ifeoma Ugonabo, Neeraja Yedlapati, John Lynn Jefferies

**Affiliations:** 1 Division of Cardiovascular Disease, University of Tennessee Health Science Center, Memphis, TN 38103, USA; 2 Division of Cardiology, University of Nevada, Las Vegas School of Medicine, Las Vegas, NV 89154, USA

Hypertrophic cardiomyopathy (HCM) is the most common genetic cardiomyopathy resulting from a mutation in one of several cardiac sarcomeric proteins. The disease is manifested by abnormal and often asymmetrical hypertrophy of the left ventricle in the absence of any secondary cardiac or systemic disease that can increase the left ventricle (LV) wall thickness, such as systemic hypertension or aortic stenosis [1,2].

Historically, HCM has been described in the literature by different names such as idiopathic hypertrophic subaortic stenosis, hypertrophic obstructive cardiomyopathy and muscular subaortic stenosis. At present, HCM has emerged as the preferred name as not all patients have left ventricular outflow tract (LVOT) obstruction [3]. HCM was thought to be a rare condition in the past but is now recognized as more common. The prevalence ranges from 1 in 200 to 1 in 500 in the general population worldwide [1,2,4]. 

Over 400 different mutations in the 11 genes encoding the proteins of the myocardial sarcomeres have been described in conjunction with HCM. These sarcomeric proteins have various structural, contractile, and regulatory functions. Ninety percent of mutations involve one of these three proteins: beta myosin heavy chain, myosin binding protein C and cardiac troponin T. Most mutations are expressed in an autosomal dominant manner which underscores the importance of screening all first-degree relatives when a diagnosis of HCM is made [4,5]. The genetic mutations in HCM cause abnormal signaling pathways in the sarcomeres resulting in excessive contractility and hyperdynamic myocardium. Histologically, myocytes are seen to be arranged in disorganized patterns (cellular disarray) in a background of myocardial fibrosis [6].

The clinical presentation is very heterogenous. Many patients have no symptoms at all and are identified incidentally or during screening. Patients with symptoms commonly have dyspnea, angina, palpitations and syncope. These symptoms can be contributed to LVOT obstruction, diastolic dysfunction, arrhythmias, and myocardial ischemia [4].

One of the hallmarks of HCM is the presence of a dynamic systolic pressure gradient in the LVOT resulting from contact between the mitral valve leaflets and the hypertrophied interventricular septum. About one-third of patients have LVOT obstruction at rest, one-third have LVOT obstruction on provocation and the remaining have nonobstructive disease with no gradient at all [4,5].

Patients with HCM are prone to brady and tachyarrhythmias, heart failure and sudden cardiac death (SCD). HCM is the most frequent cause of SCD in young people. Patients at a higher risk of SCD are considered as candidates for implantable cardioverter defibrillator therapy [2,5,7].

The diagnosis of HCM is confirmed by imaging with transthoracic echocardiography (TTE) and complemented by cardiac magnetic resonance (CMR) imaging. TTE is essential in evaluating the pattern and extent of LV hypertrophy, LV diastolic dysfunction, mitral valve systolic anterior motion, severity and mechanism of mitral regurgitation, and LVOT obstruction gradient [1,4,8].

Based on the 2020 American Heart Association/American College of Cardiology guidelines, the management of HCM patients depends on the presence of symptoms. Treatment of symptomatic HCM patients with normal LV ejection fraction (LVEF) consists mainly of beta-blockers, non-dihydropyridine calcium channel blockers, and disopyramide. Due to their negative inotropic and chronotropic effects, they decrease LVOT gradient, prolong LV filling time, improve LV filling pressure, and provide anti-arrhythmic effect. Despite their widespread use in HCM, these conventional treatments are only backed by data derived from small clinical trials and observational studies [1]. The second line treatment for patients who fail medical management is invasive septal reduction therapies (SRT) with the goal of reducing the LVOT gradient and improving obstructive symptoms. SRT can be achieved by surgical myomectomy or alcohol septal ablation. Both are very effective invasive means of reducing LVOT gradient, improving symptoms, and reducing heart failure in the future. Both SRT procedures have a low rate of complications with a 1% mortality rate. Complications include the need for permanent pacemaker placement, ventricular septal defect, and coronary dissection specifically with alcohol ablatio [1,5].

The above-mentioned medical and surgical treatments target LVOT obstruction and symptom improvement, however, they have no effect on the underling pathophysiological process driving the disease. This has changed with the approval of Mavacamten by the FDA in April of 2022. Mavacamten (initially named as MYK-461) is the first drug that was specifically designed to treat HCM. It is a first-in-class selective inhibitor of cardiac myosin ATPase activity and is a potent negative inotrope. It inhibits the interaction between myosin and actin filaments and attenuates the hypercontractility of the sarcomere characteristic of HCM [9]. PIONEER-HCM, was a small phase-two clinical trial that studied the effect of Mavacamten in 21 patients with obstructive HCM. It demonstrated the drug’s safety profile and showed improvement in symptoms along with reduction in LVOT gradients [10].

This paved the way for the landmark phase-three clinical trial of EXPLORER-HCM. This was a randomized double blinded control multicenter study that investigated the efficacy and safety of mavacamten in symptomatic obstructive HCM. The trial included 251 adult patients with obstructive HCM, with an LVOT gradient over 50 mmHg at rest and New York Heart Association (NYHA) class II-III symptoms. Patients were randomized to mavacamten or placebo for 30 weeks. The primary outcome was a composite of either at least a 1.5 mL/kg/min increase in peak minute oxygen consumption (pVO2) with a reduction in NYHA class, or at least a 3.0 mL/kg/min increase in pVO2 with no worsening of NYHA class. The results of the trial were very encouraging with 37% of subjects in the treatment arm meeting the composite primary outcome compared to 17% of the placebo arm (*p* = 0.0005). There was a significant reduction in post-exercise LVOT gradient (*p* < 0.0001) at the end of 30 weeks. Patients who received the drug had an improvement in self-reported symptoms [11].

EXPLORER HCM CMR was a follow up study in which researchers analyzed the CMR data for patients in the EXPLORER HCM trial. There was favorable remodeling in the mavacamten arm compared to the placebo group with decrease in LV mass index. The findings of regression and alteration of structural changes of the HCM phenotype seen with mavacamten treatment have been hailed as a major breakthrough in the management of HCM [12].

The MAVERECK trial was a small exploratory study designed to assess safety and tolerability in patients with non-obstructive HCM. It was not powered to determine efficacy. Mavacamten reduced cardiac biomarkers including N-terminal pro–B-type natriuretic peptide and cardiac troponin I levels. The medication was well tolerated [13]. Further phase-three trials will be required to draw accurate conclusions in non-obstructive HCM. 

So far, the results from mavacamten trials have been very promising but long-term data for both safety and efficacy are still needed. Aficamten, a next-generation myosin inhibitor, has generated considerable interest. Aficamten has a shorter life and achieves a steady state within two weeks versus four to six weeks with mavacamten. Data from a phase-two trial for aficamten (REDWOOD-HCM) have shown a favorable safety profile and response rate in the treatment arm. [14] A phase-three trial for aficamten in obstructive HCM (SEQUOIA-HCM) is currently recruiting patients [15].

There is continuous rapid progress in the understanding of HCM with encouraging advances in treatment modalities. The recent introduction of myosin inhibitors has opened the doors for disease modifying treatment. The future appears bright for these patients with hopes of improved life expectancy and quality of life. 
